# In vitro metabolic capacity of carbohydrate degradation by intestinal microbiota of adults and pre-frail elderly

**DOI:** 10.1038/s43705-021-00065-5

**Published:** 2021-10-28

**Authors:** Ran An, Ellen Wilms, Madelon J. Logtenberg, Mara P. H. van Trijp, Henk A. Schols, Ad A. M. Masclee, Hauke Smidt, Daisy M. A. E. Jonkers, Erwin G. Zoetendal

**Affiliations:** 1 Laboratory of Microbiology, Wageningen University & Research, Wageningen, The Netherlands; 2 Division Gastroenterology-Hepatology, Department of Internal Medicine, NUTRIM School of Nutrition and Translational Research in Metabolism, Maastricht University Medical Centre+, Maastricht, The Netherlands; 3 Laboratory of Food Chemistry, Wageningen University & Research, Wageningen, The Netherlands; 4 Nutrition, Metabolism & Genomics Group, Division of Human Nutrition and Health, Wageningen University & Research, Wageningen, The Netherlands

**Keywords:** Microbial ecology, Microbiome, Microbiota

## Abstract

Globally increased life expectancy strongly triggered interest to delay the onset of frailty, which has been associated with alterations in compositional and functional characteristics of intestinal microbiota. In the current study, we used an in vitro batch incubation model to compare the metabolic capacity of the faecal microbiota of adults (*n* = 6) versus pre-frail elderly (*n* = 6) to degrade various glycosidic carbohydrates, including galacto-oligosaccharides, 2′-fucosyllactose, chicory fructo-oligosaccharides and inulin, and isomalto/malto-polysaccharides. The in vitro metabolic capacity was also compared with an in vivo GOS intervention study based on the same subjects. Analysis of 16S rRNA gene sequences and metabolites revealed distinct portions of variation in overall microbiota and metabolite composition during incubation being explained by individuality of the subjects and carbon source. In addition, the age group of the subjects also had significant impact on microbiota variation, carbohydrate degradation and metabolite production. This was accompanied by elevated increase in the relative abundance of *Bifidobacterium* in the microbiota of adults compared to that of pre-frail elderly and significantly decreased effectiveness to degrade galacto-oligosaccharides by the latter group. Altogether, the carbohydrate degradation in elderly was different compared to adults, with some carbohydrates showing decreased degradation rates. Longer interventions periods may be required to enhance bifidobacterial abundance in the microbiota of pre-frail elderly and thereby to obtain associated prebiotic health benefits.

## Introduction

The human intestinal tract is inhabited by a large number of microorganisms, collectively called the intestinal microbiota. The intestinal microbiota plays an important role in human health as it converts indigestible carbohydrates into metabolites, which are largely absorbed by the intestine, but it also produces essential vitamins and affects immune system development and functioning [1].

Ageing is associated with alterations in intestinal physiology and functionality, such as increased oro-caecal and colonic transit time, thereby affecting the inhabiting microorganisms [2]. Many studies have investigated the changes in faecal microbial composition during ageing by comparing the microbiota of adults and elderly [3–6]. Notably, studies investigating the intestinal microbial composition of centenarians demonstrated that parameters related to health status rather than chronological ageing per se were associated to the changes in microbiota composition during ageing [4, 7]. Further, it should be noted that age cut-off for elderly, varying from 60–80 years, is inconsistent between studies making comparative analyses between studies difficult [8]. As such, it is not surprising to have contradictory findings being reported in terms of changes in microbiota composition during ageing [9]. Nevertheless, compared to adults, the intestinal microbiota of elderly has frequently been reported to contain higher levels of streptococci and Enterobacteriaceae as well as lower levels of *Bifidobacterium*, with differences being more pronounced in frail or comorbid elderly [10]. In addition, comparative microbiota composition analysis revealed a lower level of *Bifidobacterium* in pre-frail elderly compared to healthy adults [11]. Rampelli et al. employed a metagenomic approach and revealed that the microbiota of elderly had decreased saccharolytic potential, as shown by a loss of genes involved in carbohydrate metabolism and decreased number of genes coding for enzymes involved in short chain fatty acid production, in comparison to the microbiota of young adults [12]. Nevertheless, as it has been shown that both within and across microbial groups, microorganisms demonstrate high levels of functional redundancy [13], the impact of these reduced gene numbers on intestinal function has yet to be demonstrated.

While DNA-based approaches to study the microbiota can be used to predict its functional potential, the actual activity of microorganisms depends on different biotic and abiotic conditions [14]. Moreover, microbes are very versatile and can quickly adapt to changes in their living environment [15]. Hence, DNA-based approaches do not provide information about actual metabolic activity of the microbiota towards the degradation of specific carbohydrates. Therefore, in vitro incubations under conditions simulating the intestinal tract can be used to verify functional prediction and to provide refined information regarding to functional capacity.

Given the differences in microbiota composition between elderly and younger adults, notably with respect to the number of bifidobacteria, this study aimed to compare the metabolic capacity of microbiota in adults and elderly in response to carbohydrates of different molecular structure, including galacto-oligosaccharides (GOS) [16], 2′-fucosyllactose (2′-FL) [17, 18], chicory fructo-oligosaccharides (FOS; synonym oligofructose), chicory inulin [19] and isomalto/malto-polysaccharides (IMMP) [20], which are all known to be utilized by the intestinal microbiota and often considered as bifidogenic. We hypothesized that the metabolic capacity of pre-frail elderly microbiota is lower compared to that of adults, in terms of carbohydrate degradation and metabolite production.

## Materials and methods

### Study setup

Six adults and six elderly, who were included in a previously conducted in vivo GOS intervention study [11], donated their faecal material for the current study (Fig. S1) at their first visit or at least 4 weeks after the intervention period. Each participant defecated into a stool collector (Excretas Medical BV, Enschede, the Netherlands). Directly after defecation, faecal material was divided into two portions. A small portion (~0.5 g) was frozen immediately. The remaining faeces was anoxically cryo-conserved and used as inoculum for the in vitro incubations. The viability of different microbial groups in the anoxically cryo-conserved faecal material was determined with propidium monoazide (PMA) dye. The in vitro incubations lasted for 24 h with samples collected in duplicate to compare microbiota composition, carbohydrate degradation and metabolite production between age groups (adults vs elderly). The degrading capacity for two typical bifidogenic carbohydrates, i.e., GOS and 2′-FL, was determined for the microbiota of all six adults and six elderly and compared to a non-carbohydrate control. To further extend these experiments, we also studied the degradation of other typical bifidogenic carbohydrates, i.e. FOS, inulin, and IMMP, using the faecal inocula of three adults and three elderly for which sufficient material was still available.

### Participants

The six adults (20–30 yrs) and six elderly participants (70–85 yrs) of the intervention study [11] were randomly contacted and participated in the current study, who differed significantly in age, but not in sex, BMI, alcohol consumption, smoking, medication use or dietary fibre intake (Table 1). None of the participants took acid inhibitors (e.g., proton pump inhibitors), nor antibiotics 90 days prior to the study, nor did any of the participants have a chronic disorder or major surgery, as these factors potentially could have limited participation, completion of the study, or interfered with the study outcomes. Detailed description of the inclusion and exclusion criteria has been provided previously [11]. Subject codes as shown in the results were randomly assigned in the data analysis phase and cannot be traced back to individual subjects without the specific randomization key. The study was approved by the medical Ethics Committee of the Maastricht University Medical Center+ and registered in the US National Library of Medicine (http://www.clinicaltrials.gov) with the registration number NCT03077529 [11].

**Table 1:** Characteristics of adults (n = 6) and elderly (n = 6) included in this study.

	Adults (*n* = 6)	Elderly (*n* = 6)	*P* value
Age (yrs, mean ± SD)	34.0 ± 4.2	72.8 ± 3.0	<0.001
Sex (% female)	50	50	1
BMI (kg/m^2^, mean ± SD)	24.2 ± 3.7	26.5 ± 3.0	0.277
Alcohol (%)	83.3	83.3	1
Smoking (%)	0	16.7	1
Anticoagulation use (%)	0	16.7	1
Antispasmodics (%)	0	16.7	1
Habitual dietary fibre intake (g/day)	22.4 ± 6.5	26.6 ± 2.5	0.208

Between groups, parameters, i.e., age, BMI, were tested with independent Student’s *t* test. Percentage of sex, alcohol consumption, smoking, medication use and dietary fibre intake were compared using Fisher’s exact test between groups.

*BMI* body mass index, *SD* standard deviation, *yrs* years of age.

### Dietary intake

Participants in the current study completed the dietary records on 3 consecutive days, after instructed to record their food, beverage and dietary supplement intake based on standard household units. Their nutrient intake was analyzed using the online dietary assessment tool of The Netherlands Nutrition Centre (www.voedingcentrum.nl).

### Carbohydrates

Five different carbohydrates, i.e., GOS, 2′-FL, FOS, inulin and IMMP were used as sole carbon sources in this study. GOS and the human milk oligosaccharide 2′-FL (Fucα1-2Galβ1-4Glc) were kindly provided by Friesland Campina (Amersfoort, The Netherlands). In order to mimic the actual portion of GOS utilized by intestinal microbiota, purified GOS with <3% monomers and lactose was used. Size distribution of mono- and oligomers was as follows: 2.4% degree of polymerization (DP)1, 11.3% DP2, 41.8% DP3, 25.6% DP4, 12.1% DP5, 4.6% DP6, 1.4% DP7, 0.34% DP8, 0.11% DP9. FOS and inulin were kindly provided by Sensus (Roosendaal, the Netherlands). FOS or oligofructose (Frutalose^®^ OFP) is derived from partial enzymatic hydrolysis of inulin from chicory and consisted for 92 ± 2 % of FOS (DP2-10) and for 8 ± 2 % of a mixture of fructose, glucose and sucrose. Long-chain inulin (Frutafit^®^ TEX!), termed as inulin in the current study, is also derived from chicory, comprising ≥99.5% inulin (DP2 to 60, average chain length ≥22 monomers), and ≤0.5% mixture of fructose, glucose and sucrose. The IMMP is IMMP-92 (AVEBE, Groningen, the Netherlands) which is a novel indigestible α-glycan derived from starch, with 92% of α-(1 → 6) glycosidic linkages [21].

### Faecal sample collection and storage

To store and transport freshly defecated faeces under anoxic conditions, Anaerocult^®^ A mini (Merck KGaA, Darmstadt, Germany) was activated with 10 ml nuclease-free water (Promega, Madison, WI, US), and placed next to the faeces in the stool collector before the lid was closed to create an anoxic atmosphere. Afterwards, the stool collector and two open bags of AnaeroGen (AnaeroGen^TM^ 3.5 L Sachet, Thermo Scientific, Waltham, Massachusetts, US) were put into an anoxic box (AnaeroPack™ 7.0 L Rectangular Jar, Thermo Scientific) and stored at 4 °C until transportation. Samples were transported on ice from Maastricht to Wageningen University & Research within 9 h. After arrival, the anoxic box was transported immediately into the anaerobic chamber (MK3 Workstation, Don Whitley, UK), filled with an atmosphere of 4% H_2_ and 96% N_2_. For each donated sample, every 17.5 g faeces were mixed with 7.5 g dialysate (Tritium Microbiologie, Eindhoven, the Netherlands), 35.7 g nuclease-free water and 9.8 ml glycerol. The mixed faecal slurry was transferred into a serum bottle and sealed with a butyl rubber stopper and metal crimp cap inside the anaerobic chamber, and afterwards stored at −80 °C.

### In vitro incubations

Anoxically cryo-conserved faecal inoculum was defrosted and transferred to an anaerobic chamber filled with an atmosphere of 96% N_2_ and 4% H_2_ (BACTRON 300, Shel Lab, Cornelius, Oregon, US). Using standard ileal efflux medium (Tritium Microbiologie) [20], incubation was done with one of the carbohydrates (10 mg/ml) and 10% (v/v) faecal inoculum in duplicate, while incubations without faecal inoculum or without carbohydrates, respectively, served as controls. Specifically, every 1 L of medium comprised 400 ml BCO (60 g/L casein, 60 g/L bacto peptone and 1 g/L ox bile), 16 ml salts solution (156.3 g/L di-potassium hydrogen phosphate, 281.3 g/L sodium chloride, 28.13 g/L calcium chloride dihydrate, 0.31 g/L iron (II) sulfate heptahydrate,0.63 g/L hemin porcine), 4 ml cystein.HCl solution, 0.8 ml vitamin mix (1 mg/L menadion, 2 mg/L D(+)biotine, 0.5 mg/L Vitamin B12, 10 mg/L D(+)pantothenate, 5 mg/L aminobenzoic acid, 4 mg/L thiamine HCL and 5 mg/L nicotinamide adenine dinucleotide free acid) and 100 ml MES (1 M pH 6.0). Except for the to-be-studied carbohydrates (i.e., IMMP, short chain & long chain inulin, 2′-FL and GOS), no additional carbohydrates were added in the fermentation medium. Ten ml batch incubation bottles were used in the current study, and filled with 6 ml fermentation medium. Cultures were incubated at 37 °C on a rotary shaker at 200 rpm for 24 h.

### Sample collection

Samples were collected 0, 4, 10 and 24 h after inoculation (Fig. S2). Specifically, at each time point, two incubation bottles (the duplicate) per treatment were sacrificed for sample collection. The headspace gas was sampled first to determine H_2_ and CH_4_ production. Three aliquots of 1 ml culture were then distributed into 1.5 ml Eppendorf tubes. One of these aliquots was heated at 100 °C for 5 min to determine carbohydrates in the supernatant. Afterwards, all aliquots were centrifuged at 4 °C at 18,600 rcf for 10 min. The supernatants from the other two unheated tubes were stored at −20 °C for metabolite measurement, while the remaining pellets were stored at −80 °C for microbiota analysis.

### Carbohydrate, gas, and metabolite measurements

Degradation of GOS, 2′-FL, FOS, inulin and IMMP was determined using High-Performance Anion Exchange Chromatography (HPAEC) with Pulsed Amperometric Detection (PAD). Specifically, samples taken during the incubation were diluted and centrifuged for 15 min at 18,600 rcf. Ten microlitres of supernatant was injected to an ISC5000 HPLC system (Dionex, Sunnyvale, CA, US), which was composed of a CarboPac PA‐1 column (250 mm × 2 mm ID), a CarboPac PA guard column (25 mm × 2 mm ID) and an ISC5000 ED detector (Dionex) in the PAD mode. Detailed description of gradients and dilution factors are provided in the supplementary information. The degradation and size of the large IMMP molecules was also determined using High Performance Size Exclusion Chromatography as described previously [20].

Headspace gas composition was measured using a CompactGC gas chromatograph (Global Analyser Solutions, Breda, The Netherlands), equipped with a Carboxen PDD precolumn (pressure: 200 kPa, split flow: 20 ml/min, column oven: 90 °C, valve oven: 80 °C) with a carrier gas flow of 20 ml/min and a TCD column (pressure: 200 kPa, split flow: 10 ml/min, column oven: 80 °C, valve oven: 80 °C).

Concentration of organic acids was determined by High Performance Liquid Chromatography (HPLC), using a SUGAR SH1821 column (SHODEX, Tokyo, Japan). The column was operated at 54 °C with a flow rate of 0.8 ml/min, using 0.01 N H_2_SO_4_ as eluent. The compounds were detected by an RID-20A (Shimadzu, Kyoto, Japan) refractive index detector at a temperature of 40 °C. Four hundred µl of collected supernatant was mixed with 600 µl of 10 mM DMSO in 0.01 N H_2_SO_4_, and 10 µl of this mixture was injected for analysis. All analytical measurement data were processed using Chromeleon ™ Chromatography Data System (CDS) Software (Thermo Scientific).

### Microbiota composition analysis

The microbiota composition in faecal- and batch incubation samples was determined by sequencing of barcoded 16S ribosomal RNA (rRNA) gene amplicons (details in Supplementary information). In short, total DNA was obtained from the collected pellet by repeated bead beating and purification with a Maxwell^®^ 16 Instrument (Promega, Leiden, The Netherlands). The V4 region of the 16S rRNA gene was amplified in triplicate using barcoded 515F [22] − 806R [23] primers and total bacterial DNA as template as described previously [24]. An equimolar mix of purified PCR products was sent for Illumina Hiseq2500 (2 × 150 bp) sequencing (Eurofins Genomics, Konstanz, Germany). Raw sequence data were processed using NG-Tax 1.0 with default settings [25]. Taxonomy was assigned based on SILVA database version 128 [26, 27]. A detailed description is provided in the supplementary information. The raw sequence data has been uploaded to the European Nucleotide Archive with accession number PEJEB41341. In addition, the total genomic DNA was used for total bacterial quantification as it has been described earlier [11].

### Viability measurements

The fraction of viable microbes in the anoxically cryo-conserved faeces was determined with PMA dye, a photoreactive dsDNA-binding dye that only penetrates the envelop of dead cells [28]. Briefly, 1 ml of anoxically cryo-conserved faecal inoculum from each donor was mixed with 2.5 µl of 20 mM PMA dye (Biotium, Inc., Fremont, CA, USA), and incubated at room temperature for 5 min in the dark, followed by treatment with a PMA-Lite^TM^ LED photolysis device (Biotium, Inc.) for 15 min. Subsequently, samples were centrifuged at 4 °C for 10 min (1500 rcf). The pellet was used for microbiota composition analysis.

### Statistical analysis

All statistical analyses were conducted in R (R-3.6.3). Relative abundance of microbial taxa was calculated based on 16S rRNA gene sequence read counts. The microbial diversity (Inverse Simpson) and richness (Phylogenetic Diversity) were calculated based on amplicon sequence variants, which were also used to calculate distance matrices. Permutational multivariate analysis of variance (PERMANOVA) was performed based on weighted and unweighted UniFrac distance matrices. Principal coordinate analysis was used to visualize the microbiota composition variation between samples [29]. Microbiota variation partitioning was assessed by fitting environmental variables (i.e., age group, sampling time point and type of carbohydrate) to weighted and unweighted UniFrac distance matrices, using the *adonis* function in the vegan package [30]. To compare and contrast alterations in microbiota composition with different carbohydrates versus non-carbohydrate control during the incubation, we used principal response curve analysis to identify genera which fit best (weights > 0.05) to explain the observed difference, using the *prc* function in the vegan package [30]. As for the metabolite data, redundancy analysis (RDA) in combination with Monte Carlo permutation was performed to assess to what extent explanatory variables, i.e., incubation time, subject- and carbohydrate-specificity, could explain the overall variation in metabolite data, using the *rda* function in the vegan package [30]. To assess the effect of age group (adult vs elderly) on the degradation of carbohydrates/concentration of metabolites during incubation, we analyzed the data using two-way mixed ANOVA, with one between-subjects factor (age group) and one within-subjects factor (incubation time), using the *anova_test* function in the rstatix package [31]. False discovery rate (FDR) correction according to the Benjamini–Hochberg procedure was applied for multiple testing when applicable. A corrected *P* value < 0.05 was considered to indicate significant difference.

## Results

### Metabolite production from carbohydrate incubation

**Fig. 1:**
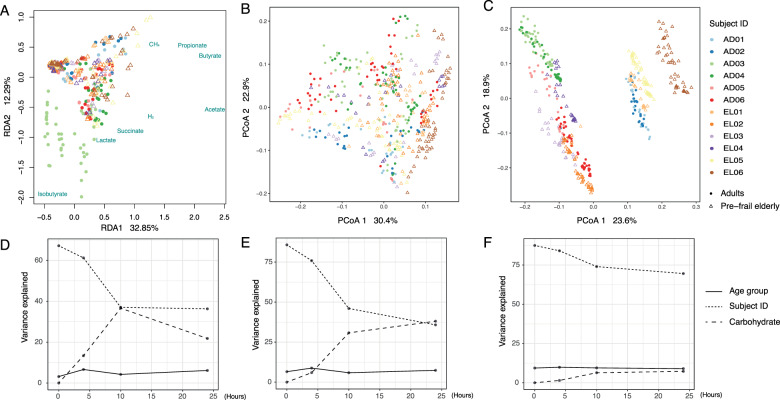
Microbiota and metabolite variation in the dataset. **A** RDA based on total metabolite data. PCoA based on **B** weighted UniFrac and **C** unweighted UniFrac distance matrices of all incubation samples. **D** variation in metabolites that can be explained by age group, subjects or carbohydrates at 0, 4, 10 and 24 h (**E**, **F**) Variation in microbiota composition that can be explained by age group, subjects or carbohydrates, based on (**E**) weighted and (**F**) unweighted UniFrac distance matrices at 0, 4, 10 and 24 h. Both duplicate samples were included for the analysis and demonstration. AD adult, EL elderly, PCoA principal coordinate analysis, RDA redundancy analysis.

**Fig. 2:**
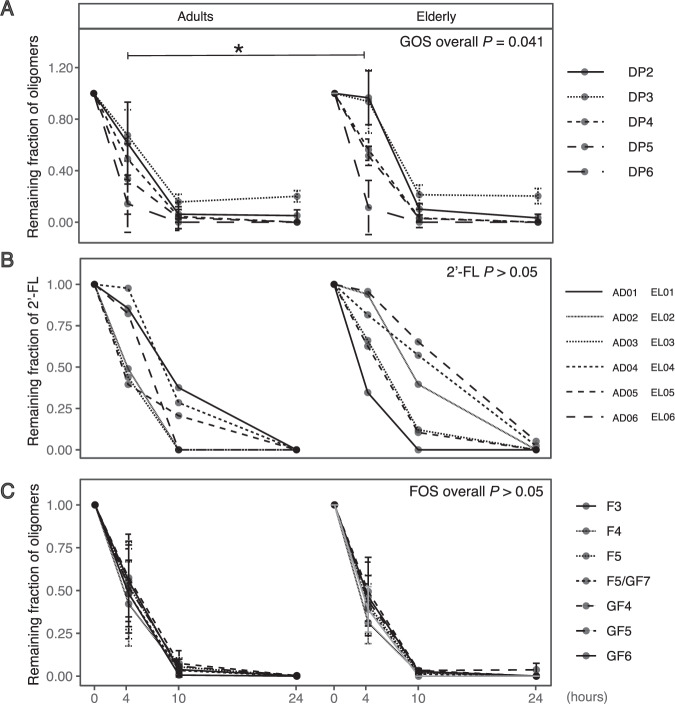
Degradation kinetics of different carbohydrates. **A** GOS, **B** 2′-FL and **C** FOS. Data are expressed as fraction of residual substrate as compared to the initial concentration of oligosaccharides or 2′-FL. Concentrations per DP in initial GOS and FOS were set to 1.0. Mean ±  SD are shown (**A** and **C**). Error bar was included to demonstrate the variability between subjects. In (**B**) no error bar was added as individual data (per subject) was shown. Individual data for **A** and **C** are available in Figs. S9 and S10. AD adult, EL elderly, DP degree of polymerization, GOS galacto-oligosaccharides, FOS fructo-oligosaccharides, 2′-FL 2′-fucosyllactose, F fructose, G glucose, SD standard deviation.

In vitro batch incubations were used to assess the metabolic capacity of the faecal microbiota of adults and elderly towards different carbohydrates. RDA revealed significant contribution of age group (2.16%), incubation time (34.66%), subject identity (16.31%) and type of carbohydrate (8.65%) to the overall variation in metabolite data (Fig. 1A), in line with what was observed for microbiota composition (Fig. 1B, C). RDA performed separately for the different time points showed that age group (3.17–6.63%) contributed to the variation in metabolite data throughout the incubation period (Fig. 1D). Over time the relative contribution of subject identity to explaining metabolite variation decreased, while contribution of carbohydrates increased (Fig. 1D), which is in line with changes in microbiota composition (Fig. 1E, F). Nevertheless, from 10 to 24 h, variation explained by carbohydrate decreased (Fig. 1D), which may be attributed to the depletion of some carbohydrates such as 2′-FL and FOS after 10 h (Fig. 2). Although buffered with MES, we observed a slight decrease of pH over time from approximately 6.4–5 as a result of carbohydrate fermentation, with slightly higher rates of acidification for FOS and IMMP for some subjects (Fig. S3). The incubation without additional carbohydrates added (i.e., the control in Fig. S3) showed a slight increase of pH over time due to the utilization of protein and subsequent production of ammonia.

Zooming in on the effect of age group on the concentration of the different metabolites (Table 2), for the GOS incubation, the concentrations of propionate and butyrate differed significantly (*P* < 0.001 and *P* = 0.048, respectively) between age groups, with significantly higher concentrations of propionate and butyrate in elderly compared to those in adults 24 h after inoculation. In contrast, the concentration of acetate did not differ significantly between adults and elderly.

**Table 2:** Concentration of metabolites (mM) during the incubation of GOS and 2′-FL by faecal inocula from elderly and adults.

Incubation time (h)		Elderly (*n* = 6)				Adults (*n* = 6)				Age group × incubation time (*P* value)
		0	4	10	24	0	4	10	24	
GOS	Acetate	1.36 [0.42,1.79]	7.44 [6.10,10.82]	45.48 [36.70,61.66]	43.83 [37.61,69.16]	1.34 [1.21,1.47]	13.83 [7.19,25.19]	55.26 [52.84,60.22]	53.45 [40.78,60.54]	0.519
	Propionate	**2.52 [0.00,2.77]**	**4.03 [2.87,5.16]**	**9.48 [6.05,11.71]**	16.84 [15.37,20.41]*	**2.24 [2.20,2.50]**	**3.99 [2.60,5.41]**	**7.13 [5.14,10.18]**	**10.74 [9.55,13.11]**	**0**
	Butyrate	**0.00 [0.00,0.11]**	**1.62 [0.45,2.97]**	**17.28 [7.53,24.25]**	47.77 [43.31,52.53]*	**0.00 [0.00,0.00]**	**2.01 [0.75,3.68]**	**8.11 [4.67,18.10]**	**28.45 [13.94,47.14]**	**0.048**
	Lactate	0.63 [0.43,0.75]	2.81 [2.48,3.23]	14.19 [4.74,25.73]	0.43 [0.00,7.12]	0.62 [0.43,0.74]	5.80 [3.47,11.11]	23.21 [19.00,26.22]	9.56 [0.80,20.16]	0.519
	Succinate	0.56 [0.46,0.66]	1.67 [1.39,2.02]	5.77 [3.90,8.40]	0.00 [0.00,1.75]	0.55 [0.00,1.19]	1.83 [0.77,3.09]	1.51 [0.46,6.98]	0.51 [0.00,7.80]	0.173
	Isobutyrate	0.00 [0.00,0.00]	0.00 [0.00,0.00]	0.00 [0.00,0.00]	0.00 [0.00,0.00]	0.00 [0.00,0.00]	0.00 [0.00,0.00]	0.00 [0.00,0.00]	0.00 [0.00,0.00]	0.259
	CH_4_	0.00 [0.00,0.00]	0.20 [0.00,0.47]	0.58 [0.00,1.52]	1.02 [0.00,4.01]	0.00 [0.00,0.00]	0.00 [0.00,0.32]	0.00 [0.00,0.91]	0.00 [0.00,2.66]	0.63
	H_2_	0.00 [0.00,0.00]	1.07 [0.95,1.23]	9.45 [8.63,12.26]	8.99 [0.02,12.58]	0.00 [0.00,0.00]	2.06 [0.72,2.73]	8.67 [3.84,12.87]	6.75 [0.04,9.90]	0.63
2′-FL	Acetate	1.54 [0.87,1.73]	7.24 [5.66,14.87]*	40.28 [31.99,47.24]*	62.57 [43.58,68.36]	1.61 [1.12,1.77]	**14.28 [7.82,22.97]**	**55.46 [46.64,59.74]**	57.14 [52.16,60.48]	0.148
	Propionate	2.80 [0.00,2.99]	4.19 [2.09,5.78]	11.89 [10.58,12.83]	25.08 [18.72,30.00]	2.15 [0.80,2.51]	4.76 [3.46,5.43]	11.71 [10.29,15.15]	23.84 [19.68,26.57]	0.519
	Butyrate	**0.00 [0.00,0.00]**	**1.66 [0.52,3.84]**	**12.02 [9.32,16.52]**	44.58 [41.30,51.62]*	**0.00 [0.00,0.07]**	**2.19 [0.85,4.36]**	**12.63 [7.93,21.33]**	**31.10 [27.12,48.71]**	**0.048**
	Lactate	0.67 [0.59,0.75]	3.05 [2.08,3.86]	2.97 [0.71,7.31]	0.00 [0.00,0.75]	0.35 [0.23,0.67]	4.45 [3.37,7.14]	3.00 [0.75,7.25]	0.00 [0.00,0.74]	0.519
	Succinate	0.29 [0.00,0.73]	1.69 [1.53,2.71]	2.52 [1.08,4.33]	0.50 [0.00,1.70]	0.65 [0.50,0.98]	2.69 [0.77,3.00]	1.33 [0.00,6.47]	0.00 [0.00,5.31]	0.884
	Isobutyrate	0.00 [0.00,0.00]	0.00 [0.00,0.00]	0.00 [0.00,0.00]	0.00 [0.00,0.00]	0.00 [0.00,0.00]	0.00 [0.00,0.00]	0.00 [0.00,0.00]	0.00 [0.00,0.00]	0.277
	CH_4_	0.00 [0.00,0.00]	0.18 [0.00,0.53]	0.65 [0.00,1.92]	0.81 [0.00,2.80]	0.00 [0.00,0.00]	0.00 [0.00,0.43]	0.00 [0.00,0.93]	0.00 [0.00,3.22]	0.63
	H_2_	0.00 [0.00,0.00]	0.88 [0.68,1.40]	6.16 [3.54,8.71]	5.59 [0.05,8.39]	0.00 [0.00,0.00]	1.90 [0.72,2.73]	8.07 [5.33,9.70]	9.10 [4.95,15.67]	0.519

Data are expressed as median [Q1, Q3] (IQR). Changes in the concentration of each metabolite over time were analyzed using two-way mixed ANOVA, with one between-subject factor (age group) and one within-subject factor (incubation time), to uncover the effect of age group (adult vs elderly) over the incubation time period. *P* values were corrected for multiple testing by FDR according to Benjamini–Hochberg procedure.

Significant differences between age groups at each time point were highlighted as bold text.

*IQR* interquartile range.

*Significant between age groups at corresponding time point.

In response to 2′-FL, the concentration of butyrate differed significantly between age groups (*P* = 0.048), with significantly higher concentration of butyrate at 24 h in elderly, compared to that in adults (Table 2). Besides, the concentration of acetate was significantly lower in elderly at 4 h (*P* = 0.049) and 10 h (*P* = 0.003) compared to that in adults.

As for FOS, the concentrations of propionate and butyrate were significantly higher in elderly after 24 h of incubation compared to those in adults (Table 3). Concurrently, the concentration of acetate did not differ between elderly and adults.

**Table 3:** Concentration of metabolites (mM) during the incubation of FOS, inulin and IMMP by faecal inocula from elderly and adults.

Incubation time (h)		Elderly (*n* = 3)				Adults (*n* = 3)				Age group × incubation time (*P* value)
		0	4	10	24	0	4	10	24	
FOS	Acetate	0.77 [0.16,1.24]	8.34 [6.21,8.86]	29.38 [23.85,64.55]	33.74 [22.24,51.03]	1.05 [0.86,1.33]	8.31 [7.53,19.11]	54.17 [52.44,57.27]	49.62 [36.91,59.28]	0.469
	Propionate	2.65 [0.61,3.20]	3.83 [2.32,4.47]	6.03 [5.62,7.14]	11.01 [10.03,15.75]*	2.51 [2.31,2.53]	3.93 [3.15,4.70]	6.77 [4.38,7.91]	**7.63 [5.06,10.53]**	0.171
	Butyrate	0.00 [0.00,0.00]	0.50 [0.12,2.53]	14.29 [5.57,27.58]	41.36 [27.00,43.71]*	0.20 [0.00,0.43]	1.36 [1.00,2.84]	8.96 [5.09,14.09]	**8.78 [4.93,26.66]**	0.136
	Lactate	0.69 [0.54,0.91]	2.58 [1.72,2.72]	27.40 [7.98,37.34]	0.00 [0.00,13.66]	0.50 [0.27,0.61]	3.54 [3.05,8.60]	32.38 [21.82,34.21]	30.20 [7.12,36.30]	0.255
	Succinate	0.99 [0.71,1.27]	2.01 [1.78,2.59]	6.06 [5.55,8.94]	2.65 [0.99,5.57]	0.60 [0.14,1.30]	3.30 [1.14,4.82]	7.82 [2.19,10.64]	8.33 [2.92,10.13]	0.293
	Isobutyrate	0.00 [0.00,0.00]	0.00 [0.00,0.00]	0.00 [0.00,0.00]	0.00 [0.00,0.00]	0.00 [0.00,0.45]	0.00 [0.00,0.75]	0.00 [0.00,0.90]	0.00 [0.00,0.50]	0.208
	CH_4_	0.00 [0.00,0.00]	0.40 [0.09,0.54]	1.31 [0.30,1.45]	1.55 [0.31,7.79]	0.00 [0.00,0.00]	0.00 [0.00,0.00]	0.00 [0.00,0.00]	0.00 [0.00,0.00]	0.208
	H_2_	0.00 [0.00,0.00]	1.68 [0.79,2.26]	17.92 [7.51,22.45]	8.05 [1.96,13.25]	0.00 [0.00,0.00]	1.76 [1.16,1.93]	5.75 [5.27,14.40]	7.38 [6.55,13.13]	0.32
Inulin	Acetate	0.00 [0.00,0.88]	5.70 [5.38,6.14]	33.81 [29.39,34.34]	37.69 [34.28,55.32]	1.11 [0.89,1.48]	5.64 [5.50,21.88]	53.37 [35.64,57.52]	50.91 [48.09,54.55]	0.293
	Propionate	2.49 [0.58,2.95]	2.69 [2.20,3.89]	8.54 [8.31,9.99]	17.53 [15.05,30.01]	2.33 [2.28,2.65]	4.05 [3.26,4.17]	8.89 [5.47,12.90]	11.56 [6.88,16.43]	0.136
	Butyrate	0.00 [0.00,0.00]	0.50 [0.00,1.25]	30.08 [18.31,30.71]	52.44 [49.71,62.14]	0.00 [0.00,0.00]	1.51 [1.12,3.30]	17.74 [14.13,27.62]	42.21 [39.61,43.39]	0.195
	Lactate	0.67 [0.58,0.76]	1.18 [1.02,1.97]	2.05 [1.66,3.32]	0.00 [0.00,0.00]	0.46 [0.10,0.96]	1.59 [1.18,6.49]	8.11 [3.64,8.91]	0.45 [0.37,1.19]	0.208
	Succinate	0.60 [0.41,1.24]	1.19 [1.09,1.70]	7.64 [7.37,9.73]	6.53 [3.89,9.78]	1.28 [0.32,1.38]	2.45 [0.59,4.51]	6.90 [2.00,10.79]	7.04 [1.60,11.49]	0.439
	Isobutyrate	0.00 [0.00,0.00]	0.00 [0.00,0.00]	0.00 [0.00,0.00]	0.00 [0.00,0.00]	0.00 [0.00,0.63]	0.00 [0.00,1.01]	0.00 [0.00,0.87]	0.00 [0.00,0.69]	0.317
	CH_4_	0.00 [0.00,0.00]	0.30 [0.07,0.70]*	1.29 [0.32,3.52]*	6.50 [1.24,9.00] *	0.00 [0.00,0.00]	**0.00 [0.00,0.00]**	**0.00 [0.00,0.00]**	**0.00 [0.00,0.00]**	0.102
	H_2_	0.00 [0.00,0.00]	0.83 [0.45,1.74]	20.34 [9.12,25.20]	1.66 [0.09,12.31]	0.00 [0.00,0.00]	1.51 [0.91,1.98]	9.76 [6.07,17.08]	11.12 [9.29,12.62]	0.208
IMMP	Acetate	0.00 [0.00,0.85]	5.38 [5.25,5.97]	32.96 [30.61,37.44]	60.34 [52.14,68.05]	1.10 [0.78,1.23]	6.45 [6.10,7.63]	35.55 [32.04,38.50]	58.93 [57.45,61.55]	0.676
	Propionate	2.49 [0.59,2.94]	4.51 [2.72,5.21]	15.76 [13.67,16.71]	28.53 [24.93,31.53]	2.62 [2.54,2.68]	4.69 [3.69,5.20]	11.90 [8.38,18.93]	18.10 [8.71,34.30]	0.342
	Butyrate	0.00 [0.00,0.00]	0.00 [0.00,1.43]	8.41 [5.87,10.42]	29.37 [26.96,41.61]*	0.00 [0.00,0.38]	1.22 [0.69,2.04]	5.79 [5.59,7.02]	**20.84 [15.46,23.72]**	0.08
	Lactate	0.73 [0.59,0.82]	1.02 [0.86,1.17]	0.06 [0.00,0.57]	0.00 [0.00,0.00]	0.32 [0.24,0.53]	1.14 [0.52,1.64]	0.00 [0.00,0.16]	0.00 [0.00,0.00]	0.469
	Succinate	1.26 [0.84,1.46]	2.18 [1.28,2.43]	9.20 [8.37,20.06]	10.34 [8.63,21.64]	0.97 [0.69,1.28]	2.84 [1.03,3.50]	22.61 [11.67,24.55]	28.07 [14.28,32.11]	0.317
	Isobutyrate	0.00 [0.00,0.00]	0.00 [0.00,0.00]	0.00 [0.00,0.00]	0.00 [0.00,0.00]	0.00 [0.00,0.00]	0.00 [0.00,1.50]	0.00 [0.00,0.60]	0.00 [0.00,0.00]	0.223
	CH_4_	0.00 [0.00,0.00]	0.37 [0.15,0.55]*	1.32 [0.29,1.77]*	2.02 [0.56,2.08]*	0.00 [0.00,0.00]	**0.02 [0.00,0.07]**	**0.00 [0.00,0.07]**	**0.00 [0.00,0.06]**	0.08
	H_2_	0.00 [0.00,0.00]	0.17 [0.11,0.68]	0.17 [0.09,0.24]	0.42 [0.13,1.09]	0.00 [0.00,0.00]	0.67 [0.59,1.93]	0.40 [0.36,1.54]	0.09 [0.05,0.63]	0.08

Data are expressed as median [Q1, Q3] (IQR). Changes in the concentration of each metabolites over time were analyzed using two-way mixed ANOVA, with one between-subjects’ factor (age group) and one within subject factor (incubation time), to uncover the effect of age group (adult vs elderly) over the incubation time period. P-values were corrected for multiple testing by FDR according to Benjamini–Hochberg procedure.

Significant differences between age groups at each time point were highlighted as bold text.

*IQR* interquatile range.

*Significant between age groups at corresponding time point.

For incubations with inulin and IMMP, the concentration of CH_4_ was significantly higher in elderly at 4, 10 and 24 h compared to that of adults. In addition, the concentration of butyrate at 24 h was significantly higher in elderly compared to that in adults when IMMP was used.

For none of the carbohydrates, incubations with microbiota of adults and elderly differed significantly in the concentration of lactate, succinate or isobutyrate (Tables 2 and 3). However, compared to the other carbohydrates, the concentration of succinate was higher in response to IMMP (Tables 2 and 3), which coincided with predominance of *Bacteroides* in the microbiota (Figs. S4E, S5E, S6B and S7), indicating succinate as the main product of IMMP utilization by *Bacteroides*. Furthermore, the faecal microbiota of only two out of six adults and three out of six elderly demonstrated production of CH_4_, in line with detection of methanogens, i.e., Methanobrevibacter (Fig. S8).

### Degradation kinetics of GOS, FOS, 2′-FL, inulin and IMMP during incubation

Considering all DPs of GOS as a whole, the microbiota of elderly was significantly slower in GOS degradation compared to the microbiota of adults (*P* = 0.041, Fig. 2A, DP distribution in Fig. S9). Zooming in on specific DPs, the microbiota of elderly was especially slower in the degradation of DP2 (*P* = 0.047) and DP3 (*P* = 0.068), compared to the microbiota of adults (Fig. 2A).

As for 2′-FL, the microbiota of adults and elderly did not differ significantly in its degradation (Fig. 2B), although subjects with lower abundance/no faecal *Bifidobacterium* at the start and during the incubation (i.e., EL02 and EL06, Fig. S6A) were remarkably slower in 2′-FL degradation, as compared to subjects with higher relative abundance of *Bifidobacterium*, like AD03 and AD05 (Fig. S6A).

FOS is composed of fructose (F) oligosaccharides with or without a terminal glucose (G) residue. Specifically, in the current study, FOS contained 2.1% F, 1.8% F_2_, 3.4% GF, 2.8% GF_2_, 6.0% GF_3_, 27.5% F_3_, 10.0% GF_4_, 21.4% F_4_, 7.6% GF_5_, 8.9% F_5_, 2.9% GF_6_ (Fig. S10). In contrast to GOS, the microbiota of adults and elderly did not differ significantly in FOS degradation when considering all oligosaccharides as a whole (*P* > 0.05), nor after zooming in on specific oligosaccharides (Fig. 2C). In addition, degradation of FOS coincided with formation of fructose mono- (F) and dimer (F2) after 4 h of incubation, which was used quickly after 10 h of incubation, and completely depleted by 24 h (Table S1).

**Fig. 3:**
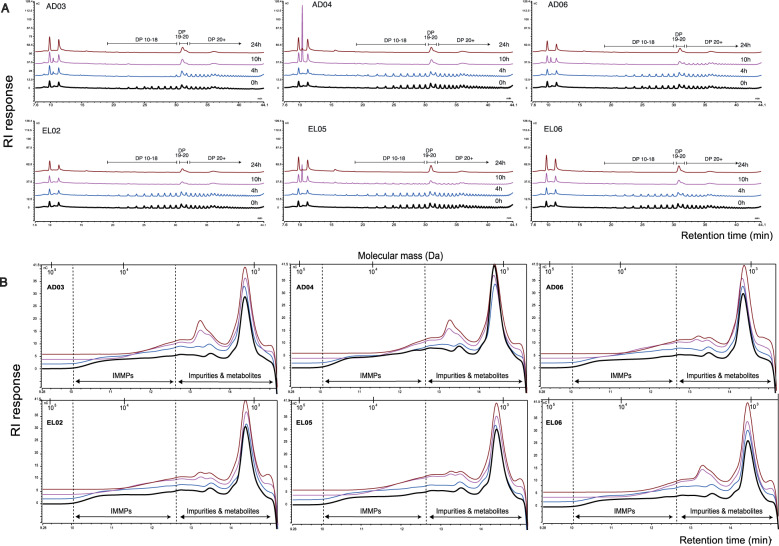
Degradation kinetics of Inulin and IMMP. **A** HPAEC elution patterns of Inulin. **B** HPSEC patterns of IMMP before and after incubation with faecal microbiota of three adults and three elderly. AD adult, EL elderly, DP degree of polymerization, IMMP isomalto/malto-polysaccharides. Incubation lasted for 24 h. Samples were taken at 0 h (Black line), 4 h (Blue line), 10 h (Purple line) and 24 h (Brown line). As duplicate samples demonstrated very high reproducibility, hereby only the chromatography elution pattern of one sample (out of the duplicate) is shown.

As compared to GOS, FOS and 2′-FL, inulin (DP2-60, distribution of DPs in Fig. S11) and IMMP are composed of longer chains, the degradation of which is visualized by HPAEC and HPSEC elution chromatograms (Fig. 3). Specifically, the inulin degradation kinetics differed between subjects (Fig. 3A). For example, the degradation of inulin was fastest by the microbiota of subject AD03 and slowest by that of AD06. In addition, inulin degradation was nearly completed after 24 h of incubation, whereas degradation of FOS only took approximately 10 h (Figs. 2C and 3A). Moreover, shorter DPs from inulin were more quickly degraded than the longer ones by the microbiota of some subjects.

The molecular weight of IMMP used in the current study ranged from 4.84 to 36.60 kDa (Fig. 3B). IMMP was not degraded after 4 h of incubation by the microbiota of any of the subjects. After 10 h, a clear shift in molecular weight of IMMP was observed, being most pronounced in AD03, AD04 and EL06. The degradation of IMMP coincided with the formation of oligosaccharides after 10 h of incubation for all subjects (Fig. S12). The concentration of these oligosaccharides was considerably higher in case of EL05 and EL06, compared to the incubations with faeces obtained from other subjects. After 24 h, the big shoulder containing large IMMP molecules (6.10–36.60 kDa, Fig. 3B) and newly formed IMMP derived oligosaccharides had nearly disappeared for all subjects (Fig. S12).

### Effect of transport and storage on the viability of the microbiota in faecal samples

PERMANOVA based on weighted and unweighted UniFrac distance matrices did not reveal significant differences between the microbiota of directly frozen faeces and that of cryo-conserved faecal inocula with or without PMA treatment (Fig. S13A, B). Although the relative abundance of some bacterial groups (Table S2), such as Bacteroidaceae and Prevotellaceae (Fig. S13C), differed in some subjects, visually reflected in larger weighted as compared to unweighted distances, comparative analysis did not show significant differences in these microbial groups. Quantification of total 16S rRNA gene copy numbers demonstrated lower fraction of microbes in the PMA treated samples (Table S3), which is in line with a previous study reporting that up to one-third of microbes in the faeces consist of dead cells [32]. Although acknowledging the limitation of the PMA analyses [33], overall, our results demonstrated a good recovery of microbes with the anoxic cryo-conservation protocol used to transport and store the samples for incubation studies.

### Changes in microbiota composition in presence of different carbohydrates

16S rRNA gene sequencing and subsequent analyses were done on duplicate samples, which show high sequencing reproducibility with Pearson similarity index 0.9880[0.9740,0.9953] (median and interquartile range) (Table S4). PERMANOVA using weighted UniFrac distances revealed significant contribution of age group (4.87%), subject identity (32.35%), type of carbohydrate (10.43%) and sampling time point (18.45%) to the overall microbiota variation (Fig. 1B), while the contribution of these factors was 8.06%, 64.50%, 1.86% and 8.44%, respectively, based on unweighted UniFrac distances (Fig. 1C).

Variation partitioning per sampling time point demonstrated significant contribution of age group (*P* < 0.01) throughout the incubation period, although its contribution remained low (5.87–9.86%) compared to that of subject identity and carbohydrate (Fig. 1E, F). Based on weighted and unweighted UniFrac distance matrices, variation explained by subject identity was highest at 0 h (85.76% and 87.47%, respectively) and decreased gradually towards 35.78% or 69.56%, respectively, over time. In contrast, the type of carbohydrate did not contribute to the microbiota variation at the start of the incubation as expected, whereas with the progress of incubation, its contribution increased to 38.07% (weighted UniFrac) or 7.26% (unweighted UniFrac) at the end of the incubation. Moreover, during the incubation, the microbial diversity and richness decreased significantly both in the microbiota of adults and that of elderly (Fig. S14 and Table S5) indicative for a selective stimulation exerted by the different carbohydrates during the incubation.

During the incubation, in comparison to non-carbohydrate controls, a large number of genera changed in their relative abundance in response to different carbohydrates, while altered genera differed between carbohydrates (Figs. S4 and S5). Among other genera, *Bifidobacterium* relative abundance increased the most in response to FOS, 2′-FL, GOS and inulin. Notably, compared to other carbohydrates, the increase in the relative abundance of *Bifidobacterium* was largest in response to GOS (Figs. S4F and S5F). This alteration was in general more pronounced in the microbiota of adults than that of elderly, with few exceptions (Fig. S6A). Interestingly, in response to inulin, the microbiota of adults and elderly changed differently, i.e., inulin supplementation lifted the relative abundance of *Bifidobacterium* in the adults (Fig. S4D), while it promoted the relative abundance of *Blautia* in the pre-frail elderly (Fig. S5D). Moreover, in the presence of IMMP, the relative abundance of *Bacteroides* increased over time both in adults and elderly (Figs. S4E, S5E and S6B). Finally, the relative abundance of *Dorea* and *Coprococcus* 3 mainly significantly increased in the non-carbohydrate controls, except for the microbiota of EL06, which also showed an increased level of *Dorea* in the presence of all five studied carbohydrates (Figs. S4, S5, and S6C).

**Fig. 4:**
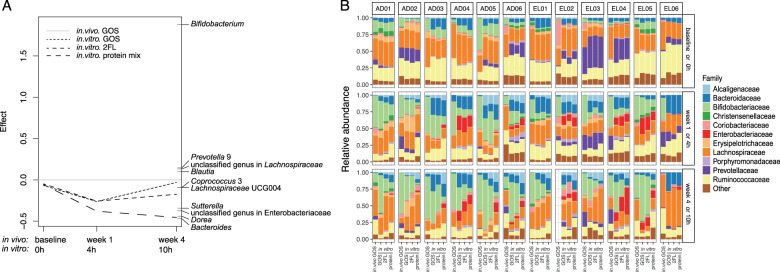
Comparative analysis between in vitro and in vivo [11] including both adults and elderly. **A** First axis of the principal response curve showing alterations in microbial composition over time in response to GOS in vivo and in response to non-carbohydrate control (protein mix) and carbohydrates (GOS or 2′-FL) in vitro, while taking in vivo GOS intervention as reference. Both duplicate samples were included for the analysis. Genera for which the model best explained the observed variation between reference and treatments (weights > 0.05) are shown on the right side of the figure. **B** Relative abundance of different bacterial families (top 12, ranked based on the average relative abundance across the entire dataset) in the microbiota of six adults and six elderly. Averaged relative abundance of the duplicate samples was used here (**B**) for visibility. Top 12 microbial families are listed in the legend. Other families are summarized as “Other”. Each column represents the corresponding type of sample from one subject. Samples collected at 24 h were excluded from this comparative analysis as some carbohydrates were completely depleted within 10 h (see Figs. 2 and 3). AD adult, EL elderly, GOS galacto-oligosaccharides, 2′-FL 2′-fucosyllactose.

To assess how well the incubation of faecal microbiota in the presence of carbohydrates in vitro reflects the in vivo observations, we compared microbiota composition and its dynamics over time between the in vitro GOS incubations and the in vivo GOS effects [11] of the same subjects (Figs. 4, S15, S16 and Table S6). Although we realize that the timelines between in vivo and in vitro are completely different, this comparison suggests that incubation of carbohydrates in vitro can to some level mimic the in vivo observations with respect to alterations in microbiota composition (Figs. 4A and S17), especially its impact on the relative abundance of *Bifidobacterium* (Fig. 4A). In two subjects, *i.e*. EL02 and EL06 (Figs. 4B and S15), the relative abundance of *Bifidobacterium* was lower or even below the detection threshold at the start of the incubation, with incongruent alterations in *Bifidobacterium* levels in response to GOS (Figs. 4B, S6A and S15). The relative abundance of *Bifidobacterium* in EL02 increased after one week of GOS supplementation, whereas in vitro only a subtle increase was observed (Figs. S15 and S16). In contrast, for EL06, the microbiota did not have detectable levels of *Bifidobacterium* at baseline both in vitro and in vivo. After 1 week of GOS intervention, the relative abundance of *Bifidobacterium* increased while, in contrast to other elderly, it completely disappeared after 4 weeks GOS supplementation (Fig. S15). Remarkably, in line with this observation, the relative abundance of *Bifidobacterium* stayed under the detection limit during the in vitro incubations with faecal inoculum from this subject (Figs. 4B and S16). Overall, after 4 weeks of GOS intervention in vivo, the microbiota was more similar to the in vitro profiles after 10 h of GOS incubation (Figs. 4A and S17), compared to that after 10 h of 2′-FL or protein incubation.

## Discussion

In the current study, we compared the metabolic capacity of faecal microbiota obtained from adults and pre-frail elderly during an in vitro incubation study in medium mimicking the intestinal lumen and using carbohydrates varying in molecular structure as carbon and energy source. We hypothesized that the microbiota of pre-frail elderly is less efficient in carbohydrate degradation and metabolite production. We found that a significant fraction of the microbiota variation and metabolite production could be explained by age group, although the different subjects and carbohydrates explained most of the variation. Our data on microbiota composition and metabolite production supported the notion that the metabolic capacity of carbohydrate degradation by the microbiota of pre-frail elderly differed from that of healthy adults, with some carbohydrates being degraded at a decreased rate in the former group.

Although the overall dietary or fibre intake and the health status of the pre-frail elderly in this study were similar to that of adults based on comorbidity, immune and oxidative stress markers [11], the lower relative abundance of *Bifidobacterium* in elderly was evident. This is in line with earlier observations that the microbiota of elderly is characterized by lower relative abundance of *Bifidobacterium* compared to that of healthy adults as recently reviewed [10]. The genomes of *Bifidobacterium* spp. contain a large number of genes encoding carbohydrate modification enzymes, such as glycosyl hydrolases, ABC transporters and the phosphoenolpyruvate-phosphotransferase system, and hence, bifidobacteria act actively in carbohydrate degradation and utilization [34]. In the current study, decreased relative abundance of *Bifidobacterium* in the microbiota of pre-frail elderly at the start of the incubation, as well as a less uniform/consistent increase in *Bifidobacterium* relative abundance during incubation compared to that in healthy adults, could have contributed to the declined efficiency in the carbohydrate degradation, especially for GOS [35], 2′-FL [36] and inulin [37], which are known as bifidogenic ingredients. Collectively, it is possible that a lower efficiency in degradation of some bifidogenic components could be a sign of changed intestinal conditions in pre-frail elderly. Nevertheless, it is hard to decipher cause-consequence relations and that our statement remains speculative. Moreover, the impact of lower *Bifidobacterium* (relative) abundance on the intestinal physiology during the ageing process remains to be further explored.

We observed differences in altered genera during the incubation of different carbohydrates. Although in line with other studied carbohydrates with respect to the observed increase in the level of *Bifidobacterium* during the incubation, the presence of IMMP was particularly associated with an increase of *Bacteroides*, which was accompanied with increased concentration of succinate in both age groups. Consistently, in addition to the increase in the relative abundance of *Bifidobacterium*, Gu et al. observed an increase in the relative abundance of *Bacteroides* and the concentration of succinate during in vitro incubation of IMMP with adult faecal microbiota [20]. Generally, only low concentrations of succinate are observed in the human intestine [38] as an intermediate in the synthesis of propionate, a common product of *Bacteroides* or Veillonellaceae through the succinate pathway [39]. In the current study, a significant increase in the relative abundance of *Bacteroides* resulted in the accumulation of succinate. As reviewed by Fernandez-Veledo et al. [40], several recent studies in humans and in mice have suggested to treat obesity and related co-morbidities via modulating the succinate level in the intestine. Hence, the IMMP studied here could be a potential candidate for intervention studies to investigate its direct and indirect impact on human health. On the other hand, although no significant difference in succinate accumulation between adults and elderly were observed, it seems that the higher succinate production in AD03, AD04 and EL06 coincided with faster acidification when compared to the other subjects. Similar observations were made for lactate production and faster acidification in the GOS and FOS incubations. These results could imply that differences in initial pH drop could play a role in subsequent microbial activity including the conversion of lactate to butyrate, which has been observed previously in in vitro study setups [41, 42]. Consistent with the observations in carbohydrate degradation, the dynamics of metabolite production over time differed significantly between adults and pre-frail elderly, in addition to the effect of carbohydrate- and subject-specificity. On one hand, this is in line with the high individuality shown in the microbiota composition. For instance, only incubations with faecal inocula obtained from subjects with detectable levels of methanogens i.e., *Methanobrevibacter*, demonstrated CH_4_ production, further reinforcing individual differences in metabolic capacity of the microbiota. On the other hand, our data also emphasized the differences in microbial composition between age groups. Compared to healthy adults, the microbiota of pre-frail elderly had lower levels of *Bifidobacterium*, and as such was slower in 2′-FL utilization and acetate production at the beginning of the incubation [43]. At later time points, compared to healthy adults, incubation with microbiota of pre-frail elderly had higher concentrations of propionate and butyrate, indicating the presence of propionate and butyrate-producing microbes like *Coprococcus* [43]. The relative abundance of the latter genus was also higher in pre-fail elderly than in adults at the start of the incubation. Some of the other carbohydrates, such as FOS and inulin, did not show significant differences in their degradation rate between the age groups. This could be attributed to the existence of FOS and/or inulin in daily diet (e.g., wheat, banana and onion). Therefore, the elderly microbiota may not need to adapt to their degradation, as such could use them to produce the main SCFAs. Interestingly, in response to inulin *Blautia* was increased in abundance in elderly versus *Bifidobacterium* in young adults. Bifidobacterial effects have been observed in elderly human intervention [44]. Perhaps in this in vitro gut model, the low levels of bifidobacteria in elderly faeces allowed metabolism of the long chains of inulin by other microbes, which has been reported [45], and in this case Blautia. Furthermore, the differences in metabolites between age groups varied for different carbohydrates, which could in part be attributed to the differences in carbohydrates’ physical and chemical properties [19], although it remains challenging to explain comprehensibly how carbohydrate properties affect microbiota composition, metabolite production, as well as the corresponding direct/indirect impact on health.

We clearly showed that all carbohydrates were utilized over time. In some cases (e.g., 2′-FL and IMMP) we also observed that some of the remaining cryoprotective agent glycerol was utilized over time. Previous studies have shown that glycerol could possibly favour some bacterial growth and corresponding metabolism [46]. Although we never saw a preference of the community for glycerol over the carbohydrate, we realize that it is a potential additional carbon source in our system, and therefore, we cannot exclude its impact on the microbiota composition and metabolite profiles in the cases in which we observed its utilization. This general drawback of cultivation experiments with microbial communities is difficult to circumvent as cryoprotection is crucial when samples are collected for in vitro cultivation experiments, in addition to the limitation of medium dependency and selectivity.

We performed the in vitro incubation of different carbohydrates using faecal inocula of a subgroup of subjects involved in a GOS intervention study. Comparative analyses revealed that the GOS intervention data best matched with data from the GOS incubation in the in vitro experiment, conferring that in vitro approaches can at some level offer a good model for in vivo observations. Moreover, we demonstrated a large contribution of inter-individual differences in microbiota composition, and microbial capacity with respect to carbohydrate degradation and metabolite production, which collectively underscored the importance of taking individual-specific differences into account in future studies.

In conclusion, the efficacy of carbohydrate degradation by the microbiota of pre-frail elderly differed from that of healthy adults, with some carbohydrates showing significantly decreased efficacy of degradation by the microbiota of pre-frail elderly. Although the pre-frail elderly showed no physical and immune decline yet [11], the lower level of *Bifidobacterium* and their lower involvement in carbohydrate degradation made us speculate that a microbial change with declined efficacy of certain prebiotic carbohydrates degradation may be expected in the microbiota of pre-frail elderly. Whether this has an impact on the progress of frailty or other health parameters in elderly and whether specific (dietary) interventions can postpone this process is speculative and could be addressed in follow-up studies.

## Supplementary information

Supplementary Information

Fig.S1

Fig.S2

Fig.S3

Fig.S4

Fig.S5

Fig.S6

Fig.S7

Fig.S8

Fig.S9

Fig.S10

Fig.S11

Fig.S12

Fig.S13

Fig.S14

Fig.S15

Fig.S16

Fig.S17

Table.S1

Table.S2

Table.S3

Table.S4

Table.S5

Table.S6
