# Supplementary material for: In vitro metabolic capacity of carbohydrate degradation by intestinal microbiota of adults and pre-frail elderly
Source: ISME Commun. 2021 Oct 28;1:61. doi: 10.1038/s43705-021-00065-5 (PMC9723549; doi:10.1038/s43705-021-00065-5)
Supplement: Supplementary file 1 — Supplementary Information [file 43705_2021_65_MOESM1_ESM.docx]

**Supplementary information**

**Gradients for the measurement of carbohydrate degradation using HPAEC-PAD**

The collected (heated) supernatants were used to study the carbohydrate degradation during the incubation. Degradation of GOS, 2’-FL, FOS, inulin and IMMP was determined using High Performance Anion Exchange Chromatography (HPAEC) with Pulsed Amperometric Detection (PAD). Specifically, samples taken during the incubation were diluted to 50 µg/ml (GOS, FOS and inulin), 5 µg/ml (2’-FL) or 1mg/ml (IMMP) of initial substrate concentration, and then centrifuged for 15 min at 15000 g. Subsequently, 10 µl of supernatant was injected to an ISC5000 HPLC system (Dionex, Sunnyvale, CA, USA), which was composed of a CarboPac PA‐1 column (250 mm × 2 mm ID), a CarboPac PA guard column (25 mm × 2 mm ID) and an ISC5000 ED detector (Dionex) in the PAD mode. The flow rate was set at 0.3 ml/min. The running solvents contained 0.1 M NaOH (A) and 1 M NaOAc in 0.1 M NaOH (B). Gradient for GOS: 0-25 min 0-25% B; 25-30 min, 25-100% B; 30-35 min, washing step with 100% B; 35-35.1 min, 100-0% B; 35.1-50 min, equilibration with 100% A; GOS peaks were annotated according to Ladirat *et el*. 2014 (1). Gradient for 2’-FL: 0-15 min 0-15% B; 15-20 min, 15-100% B; 20-25 min, washing step with 100% B; 25-25.1 min, 100-0% B; 25.1-40 min, equilibration with 100% A; Gradient for FOS: 0-35 min 0-35% B; 35-40 min, 35-100% B; 40-45 min, washing step with 100% B; 45-45.1 min, 100-0% B; 45.1-60 min, equilibration with 100% A; Gradient for inulin and IMMP: 0-40 min 0-40% B; 40-45 min, 40-100% B; 45-50 min, washing step with 100% B; 50-50.1 min, 100-0% B; 50.1-65 min, equilibration with 100% A.

**Calculation method for gas production from CompactGC to mmol/L medium**

The directly obtained result from CompactGC is the percentage of H_2_ or CH_4_ in the head space by comparing with pre-made H_2_ or CH_4_ standards. The calculation has been divided into two steps: calculation to mmol H_2_/bottle then calculate to mmol H_2_/L medium.

mmol H_2_/bottle =$(\frac{Percentage of H2 determined in the head space}{100}*4ml volume of head space)/24$

mM H_2_ in Table 2&3 = mmol H_2_/L medium = $(\frac{1000}{mmol H2/bottle}*6ml liquid volume)$

**Microbiota composition analysis**

The microbiota composition in faecal- and batch incubation samples was determined by sequencing of barcoded 16S ribosomal RNA (rRNA) gene amplicons using Illumina Hiseq2500 (2 x 150 bp). Collected pellets from 1 ml batch incubation samples or 0.25 g faeces was mixed with 350 µl Stool Transport and Recovery (STAR) buffer (Roche Diagnostics, United States) and subsequently transferred into a screw cap tube containing 0.25 g of 0.1 mm zirconia beads and 3 glass beads (diameter 2.5 mm). Samples were then subjected to repeated bead beating (3 times 5.5ms × 60 s) using the FastPrep-24™ 5G bead beating grinder and lysis system (MP Biomedicals, the Netherlands) and followed by 15 min centrifugation at 4 °C. Supernatant was collected, and the pellet was subjected to another cycle of isolation with 300 µl STAR buffer. Two-hundred-fifty µl of combined supernatants was transferred into the Maxwell® 16Tissue LEV Total RNA purification Kit Cartridge (XAS1220) and processed using the Maxwell® 16 Instrument (Promega, Leiden, The Netherlands), and ultimately eluted in 35 µl of nuclease free water. The V4 region of the 16S rRNA gene was amplified in triplicate using barcoded 515F (2) - 806R (3) primers and diluted DNA (20 ng/µl) as template with an annealing temperature of 50 °C. The PCR was performed as described previously (4). An equimolar mix of purified PCR products was sent for sequencing (Eurofins Genomics, Konstanz, Germany). Raw sequencing data was processed using NG-Tax 1.0 with default settings (5), specifically as follows: forward and reverse read length is 70bp; Classification ratio = 0.8; Minimum percentage threshold = 0.1; Number of mismatches allowed for OTU clustering = 1. Taxonomy was assigned based on SILVA database version 128 (6, 7).

**Supplementary figures legends**

**Fig.S1** Flowdiagram illustrating the setup to study the carbohydrate dagradation by the faecal microbiota of adults and pre-frail elderly. GOS, galacto-oligosaccharides; 2’-FL, 2'-fucosyllactose; IMMP, isomalto/malto-polysaccharides; FOS, fructo-oligosaccharides.

**Fig.S2** Schematic representation of sample collection during in vitro incubation for different analyses. Incubation lasted for 24 h. Samples were taken at 0 h, 4 h, 10 h and 24 h after inoculation.

**Fig.S3** Individual pH at different time points of incubation with microbiota from adults (AD) and pre-frail elderly (EL), in presence of different carbohydrates and no-carbohydrate control. Incubation lasted for 24 h. Samples were taken at 0 h, 4 h, 10 h and 24 h. AD: adult; EL: elderly. GOS: galacto-oligosaccharides. FOS: fructo-oligosaccharides. IMMP: isomalto/malto-polysaccharides. 2’-FL: 2'-fucosyllactose.

**Fig.S4** Alterations in adult microbiota composition, in response to (A) GOS, (B) 2’-FL, (C) FOS, (D) inulin, (E) IMMP and (F) all carbohydrates over time. Each section of the figure (except for F) is composed of two parts: (1) PCoA analysis based on weighted UniFrac distance (each dot represents one sample and samples are coloured per sampling time point). (2) Principal response curve, *i.e.* alterations in microbial composition compared to no-carbohydrate control. Genera (weights > 0.05) for which the model best explains the observed variation between no-carbohydrate and carbohydrate-based incubation are shown on the right side of each figure. PCoA: principal coordinate analysis. GOS: galacto-oligosaccharides. FOS: fructo-oligosaccharides. 2’-FL: 2'-fucosyllactose. IMMP: isomalto/malto-polysaccharide.

**Fig.S5** Alterations in elderly microbiota composition, in response to (A) GOS, (B) 2’-FL, (C) FOS, (D) inulin, (E) IMMP and (F) all carbohydrates over time. Each section of the figure (except for F) is composed of two parts: (1) PCoA analysis based on weighted UniFrac distance (each dot represents one sample and samples are coloured per sampling time point). (2) Principal response curve, i.e. alterations in microbial composition compared to no-carbohydrate control. Genera (weights > 0.05) for which the model best explains the observed variation between no-carbohydrate and carbohydrate-based incubation are shown on the right side of each figure. PCoA: principal coordinate analysis. GOS: galacto-oligosaccharides. FOS: fructo-oligosaccharides. 2’-FL: 2'-fucosyllactose. IMMP: isomalto/malto-polysaccharide.

**Fig.S6** Changes in the relative abundance (mean of duplicates) of (A) Bifidobacterium, (B), Bacteroides (C) and Dorea in the presence of different carbohydrates and no-carbohydrate control during the incubation. GOS: galacto-oligosaccharides. FOS: fructo-oligosaccharides. 2’-FL: 2'-fucosyllactose. AD: adult; EL: elderly.

**Fig.S7** Changes in the relative abundance of Bacteroides and concentration of succinate over the incubation time period, using faecal inoculum from each subject. AD: adult; EL: elderly

**Fig.S8** Changes in the relative abundance of Methanobrevibacter and concentration of methane over the incubation time period, using faecal inoculum from each subject. AD: adult; EL: elderly.

**Fig.S9** HPAEC elution patterns of GOS and fraction of remaining GOS of individual DPs present per subject. Within the figure on top, X-axis is the retention time. Y-axis indicates the detection signal. Higher signal (and bigger peak area) indicate larger amount of correspoonding sized molecules. Starting concentrations per DP in GOS were set to 1.0. DP2 is annotated as 2.1-2.6. DP3 is annotated as 3.1-3.9. DP4 is annotated as 4.1 and 4.2. DP5 is annotated as 5.1 and 5.2. DP6 is annotated as 6.1 and 6.2. As duplicate samples demonstrated very high reproducibility, hereby only the chromatography elution pattern of one sample (out of the duplicate) was used. DP: degree of polymerization. AD: adult; EL: elderly.

**Fig.S10** HPAEC elution patterns of FOS and fraction of remaining oligosaccharides from FOS per subject. Within the figure on top, X-axis is the retention time. Y-axis indicates the detection signal. Higher signal (and bigger peak area) indicate larger amount of correspoonding sized molecules. Starting concentrations per DP in FOS were set to 1.0. As duplicate samples demonstrated very high reproducibility, hereby only the chromatography elution pattern of one sample (out of the duplicate) was used. F: fructose, G: glucose. AD: adult; EL: elderly.

**Fig.S11** HPAEC elution patterns of FOS (blue line) and inulin (black line). X-axis is the retention time. Y-axis indicates the detection signal. Higher signal (and bigger peak area) indicate larger amount of correspoonding sized molecules. DP of the oligosaccharides is as indicated. DP: degree of polymerization.

**Fig.S12** HPAEC elution patterns of IMMP before and after fermentation using faecal microbiota of three adults and three elderly. As duplicate samples demonstrated very high reproducibility, hereby only the chromatography elution pattern of one sample *(out of the duplicate)* was used*.* AD: adult; EL: elderly; IMMP: isomalto/malto-polysaccharides. Incubation lasted for 24 h.

**Fig.S13** PCoA based on (A) weighted UniFrac and (B) unweighted UniFrac distance matrices. Samples from the same subject are linked with grey lines. (C) Relative abundance of different bacterial families (top 12, ranked based on the average relative abundance across the entire dataset) in directly frozen faeces and anoxically cryo-conserved faeces from six adults and six elderly. Top 12 microbial families are listed in the legend. Other families are summarized as “Other”. Each column represents corresponding type of sample from one subject. The anoxic cryo-conserved faeces samples are further divided into with and without PMA treatment. The empty column of EL03 is due to repeated failure in DNA isolation from this specific sample. PCoA; Principle coordinate analysis. AD: adult; EL: elderly.

**Fig.S14** Microbial richness (A) and diversity (B) at different time points of incubation with microbiota from adults and pre-frail elderly, in presence of different carbohydrates and no-carbohydrate control. Incubation lasted for 24 h. Samples were taken at 0 h, 4 h, 10 h and 24 h. AD: adult; EL: elderly. GOS: galacto-oligosaccharides. FOS: fructo-oligosaccharides. IMMP: isomalto/malto-polysaccharides. 2’-FL: 2'-fucosyllactose.

**Fig.S15** Relative abundance of different bacterial families (top 12, ranked based on the average relative abundance across the entire dataset) in the microbiota of six adults and six elderly in vitro and in vivo. Averaged relative abundance of the duplicate samples (in vitro) was used here for visibility. Top 12 microbial families are listed in the legend. Other families are summarized as “Other”. Each column represents the corresponding type of sample from one subject. Samples collected at 24 h were excluded from this comparative analysis as some carbohydrates were completely depleted within 10 h. AD: adult; EL: elderly. GOS: galacto-oligosaccharides. 2’-FL: 2'-fucosyllactose.

**Fig.S16** PCoA based on (A-C) weighted UniFrac and (D-F) unweighted UniFrac distance matrices at different time points. Incubation lasted for 24h and samples were collected 0h, 4h, 10h and 24h after the inoculation. Samples collected at 24 h were excluded from this comparative analysis as some carbohydrates were completely depleted within 10 h. GOS: galacto-oligosaccharides. 2’-FL: 2'-fucosyllactose.

**Fig.S17** Distance to in vivo samples at corresponding time points. Incubation lasted for 24h and samples were collected 0h, 4h, 10h and 24h after the inoculation. Samples collected at 24 h were excluded from this comparative analysis as some carbohydrates were completely depleted within 10 h. Distance calculation was based on the weighted UniFrac distance matrice. Larger the distance value indicates decreased similarity. AD: adult; EL: elderly. GOS: galacto-oligosaccharides. 2’-FL: 2'-fucosyllactose

**References**

1. Ladirat S, Schols H, Nauta A, Schoterman M, Schuren F, Gruppen H. *In vitro* fermentation of galacto-oligosaccharides and its specific size-fractions using non-treated and amoxicillin-treated human inoculum. Bioact. Carbohydr. Diet. Fibre. 2014;3(2):59-70.

2. Parada AE, Needham DM, Fuhrman JA. Every base matters: assessing small subunit rRNA primers for marine microbiomes with mock communities, time series and global field samples. Environ. Microbiol.. 2016;18(5):1403-1414.

3. Apprill A, McNally S, Parsons R, Weber L. Minor revision to V4 region SSU rRNA 806R gene primer greatly increases detection of SAR11 bacterioplankton. Aquat. Microb. Ecol.. 2015;75(2):129-137.

4. An R, Wilms E, Smolinska A, Hermes GD, Masclee AA, de Vos P, *et al*. Sugar beet pectin supplementation did not alter profiles of fecal microbiota and exhaled breath in healthy young adults and healthy elderly. Nutrients. 2019;11(9):2193.

5. Ramiro-Garcia J, Hermes GD, Giatsis C, Sipkema D, Zoetendal EG, Schaap PJ, et al. NG-Tax, a highly accurate and validated pipeline for analysis of 16S rRNA amplicons from complex biomes. F1000Research. 2016;5.

6. Quast C, Pruesse E, Yilmaz P, Gerken J, Schweer T, Yarza P, et al. The SILVA ribosomal RNA gene database project: improved data processing and web-based tools. Nucleic Acids Res. 2013;41(Database issue):D590-596.

7. Yilmaz P, Parfrey LW, Yarza P, Gerken J, Pruesse E, Quast C, et al. The SILVA and "All-species Living Tree Project (LTP)" taxonomic frameworks. Nucleic Acids Res. 2014;42(Database issue):D643-648.
