# Supplementary figures and images for: In vitro metabolic capacity of carbohydrate degradation by intestinal microbiota of adults and pre-frail elderly
Source: ISME Commun. 2021 Oct 28;1:61. doi: 10.1038/s43705-021-00065-5 (PMC9723549; doi:10.1038/s43705-021-00065-5)

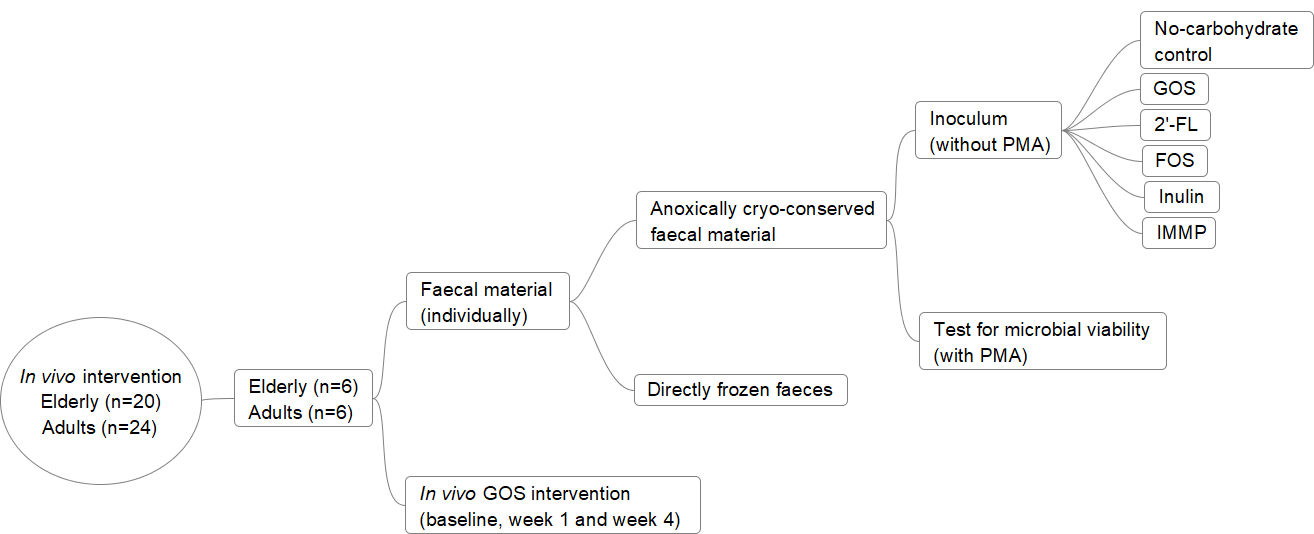

Supplement: Supplementary file 2 — Fig.S1 [file 43705_2021_65_MOESM2_ESM.png]
